# Supplementary material for: Recreational water exposures and illness outcomes at a freshwater beach in Toronto, Canada: A prospective cohort pilot study
Source: PLoS One. 2023 Jun 2;18(6):e0286584. doi: 10.1371/journal.pone.0286584 (PMC10237503; doi:10.1371/journal.pone.0286584)
Supplement: S1 File — (DOCX) [file pone.0286584.s001.docx]

STROBE Statement—Checklist of items that should be included in reports of ***cohort studies***

|  | Item No | | Recommendation | | Section | |
| --- | --- | --- | --- | --- | --- | --- |
| **Title and abstract** | 1 | | (*a*) Indicate the study’s design with a commonly used term in the title or the abstract | | Title | |
|  |  |  | (*b*) Provide in the abstract an informative and balanced summary of what was done and what was found | | Abstract | |
| Introduction | | | | | | |
| Background/rationale | 2 | | Explain the scientific background and rationale for the investigation being reported | | Introduction | |
| Objectives | 3 | | State specific objectives, including any prespecified hypotheses | | Introduction | |
| Methods | | | | | | |
| Study design | 4 | | Present key elements of study design early in the paper | | Study design and setting | |
| Setting | 5 | | Describe the setting, locations, and relevant dates, including periods of recruitment, exposure, follow-up, and data collection | | Study design and setting | |
| Participants | 6 | | (*a*) Give the eligibility criteria, and the sources and methods of selection of participants. Describe methods of follow-up | | Participation eligibility and recruitment; Data collection surveys | |
|  |  |  | (*b*) For matched studies, give matching criteria and number of exposed and unexposed | | N/a | |
| Variables | 7 | | Clearly define all outcomes, exposures, predictors, potential confounders, and effect modifiers. Give diagnostic criteria, if applicable | | Data collection surveys; Exposures and outcomes of interest | |
| Data sources/ measurement | 8* | | For each variable of interest, give sources of data and details of methods of assessment (measurement). Describe comparability of assessment methods if there is more than one group | | Exposures and outcomes of interest | |
| Bias | 9 | | Describe any efforts to address potential sources of bias | | Data collection surveys | |
| Study size | 10 | | Explain how the study size was arrived at | | Participation eligibility and recruitment | |
| Quantitative variables | 11 | | Explain how quantitative variables were handled in the analyses. If applicable, describe which groupings were chosen and why | | Data analysis | |
| Statistical methods | 12 | | (*a*) Describe all statistical methods, including those used to control for confounding | | Data analysis | |
|  |  |  | (*b*) Describe any methods used to examine subgroups and interactions | | Data analysis | |
|  |  |  | (*c*) Explain how missing data were addressed | | Exposures and outcomes of interest | |
|  |  |  | (*d*) If applicable, explain how loss to follow-up was addressed | | Data analysis | |
|  |  |  | (*e*) Describe any sensitivity analyses | | N/a | |
| Results | | | | |  | |
| Participants | 13* | | (a) Report numbers of individuals at each stage of study—eg numbers potentially eligible, examined for eligibility, confirmed eligible, included in the study, completing follow-up, and analysed | | Participant characteristics | |
|  |  |  | (b) Give reasons for non-participation at each stage | | Data not available | |
|  |  |  | (c) Consider use of a flow diagram | | N/a | |
| Descriptive data | 14* | | (a) Give characteristics of study participants (eg demographic, clinical, social) and information on exposures and potential confounders | | Participant characteristics; Tables 1-7 and Fig 1 | |
|  |  |  | (b) Indicate number of participants with missing data for each variable of interest | | Table 1 | |
|  |  |  | (c) Summarise follow-up time (eg, average and total amount) | | Exposures and outcomes of interest | |
| Outcome data | 15* | | Report numbers of outcome events or summary measures over time | | N/a | |
| Main results | | 16 | (*a*) Give unadjusted estimates and, if applicable, confounder-adjusted estimates and their precision (eg, 95% confidence interval). Make clear which confounders were adjusted for and why they were included | N/a | |  |
|  |  |  | (*b*) Report category boundaries when continuous variables were categorized | N/a | |  |
|  |  |  | (*c*) If relevant, consider translating estimates of relative risk into absolute risk for a meaningful time period | N/a | |  |
| Other analyses | | 17 | Report other analyses done—eg analyses of subgroups and interactions, and sensitivity analyses | N/a | |  |
| Discussion | | | | | |  |
| Key results | | 18 | Summarise key results with reference to study objectives | Discussion | |  |
| Limitations | | 19 | Discuss limitations of the study, taking into account sources of potential bias or imprecision. Discuss both direction and magnitude of any potential bias | Discussion | |  |
| Interpretation | | 20 | Give a cautious overall interpretation of results considering objectives, limitations, multiplicity of analyses, results from similar studies, and other relevant evidence | Discussion; Conclusion | |  |
| Generalisability | | 21 | Discuss the generalisability (external validity) of the study results | Discussion; Conclusion | |  |
| Other information | | | | | |  |
| Funding | | 22 | Give the source of funding and the role of the funders for the present study and, if applicable, for the original study on which the present article is based | Funding statement | |  |

*Give information separately for exposed and unexposed groups.
