# Supplementary material for: Recreational water exposures and illness outcomes at a freshwater beach in Toronto, Canada: A prospective cohort pilot study
Source: PLoS One. 2023 Jun 2;18(6):e0286584. doi: 10.1371/journal.pone.0286584 (PMC10237503; doi:10.1371/journal.pone.0286584)
Supplement: S2 File — (DOCX) [file pone.0286584.s002.docx]

**Investigating the Burden of Recreational Water Illness among Toronto Beachgoers:**

**Questionnaires**

**Beach Questionnaire: Household Responses**

1. Have you already participated in this study in the last 21 days?
   1. Yes (end survey)
   2. No (continue)
2. Do you currently live in Canada or the U.S.?
   1. Yes (continue)
   2. No (end survey)
3. How many members of your household are at the beach today including yourself?
   1. ________________
4. To get in touch with you for the follow-up survey in 7 days, as well as to enter you in a raffle to win an e-gift card, we will need your email address and telephone number. Please enter your email address and telephone number below:
   1. Email: _________________
   2. Telephone: ________________
5. Would you prefer that we email you a web link to the follow-up interview in 7 days, or would you prefer that we contact you by telephone to complete the interview?
   1. Email
   2. Telephone:
      1. What are the best days and times to reach you? ________________
6. What is your province or state of residence?
   1. ________________
   2. Prefer not to answer
7. What is your total annual household income?
   1. Under $20,000
   2. $20,000 to under $40,000
   3. $40,000 to under $60,000
   4. $60,000 to under $80,000
   5. $80,000 to under $100,000
   6. $100,000 to under $150,000
   7. $150,000 or above
   8. Prefer not to answer
8. What is the highest level of educational attainment among members of your household?
   1. No certificate, diploma or degree (including secondary/high school)
   2. Secondary (high) school diploma or equivalent certificate
   3. Apprenticeship or trades certificate or diploma
   4. College, CEGEP, or other non-university certificate or diploma
   5. Bachelor's degree
   6. University certificate, diploma or degree above bachelor level
   7. Prefer not to answer

**Beach Questionnaire: Individual Responses**

We have some questions about each member of your household at the beach with you today. Please answer the following questions about yourself before answering for other members of your household, answering for each person individually.

1. Please enter your first and last name:
   1. _______________
2. What is your age group?
   1. 0 to 4 years
   2. 5 to 9 years
   3. 10 to 14 years
   4. 15 to 19 years
   5. 20 to 39 years
   6. 40 to 59 years
   7. 60 years and over
3. What is your sex assigned at birth?
   1. Male
   2. Female
   3. Other, please specify: __________
   4. Prefer not to answer
4. What is your current gender identity?
   1. Boy
   2. Girl
   3. Man
   4. Women
   5. Non-binary
   6. Other, please specify: _____________
   7. Prefer not to answer
5. People often describe themselves by their race or racial background. For example, some people consider themselves “Black”, “White” or “East Asian”. Which race category best describes you? *(Check all that apply)*
   1. Arab, Middle Eastern or West Asian (e.g., Afghan, Armenian, Iranian, Lebanese, Persian, Turkish)
   2. Black (e.g., African, African-Canadian, Afro-Caribbean)
   3. East Asian (e.g., Chinese, Japanese, Korean)
   4. Indigenous (e.g., First Nations, Inuit, Métis)
   5. Latin American (e.g., Brazilian, Colombian, Cuban, Mexican, Peruvian)
   6. South Asian or Indo-Caribbean (e.g., Indian, Indo-Guyanese, Indo-Trinidadian, Pakistani, Sri Lankan)
   7. Southeast Asian (e.g., Filipino, Malaysian, Singaporean, Thai, Vietnamese)
   8. White (e.g., English, Greek, Italian, Portuguese, Russian, Slovakian)
   9. Not listed, please describe: _______________
   10. Prefer not to answer
6. Have you or any of your household members at the beach today been ill in the past 7 days with any of the following symptoms? *(Check all that apply)*
   1. Diarrhea (≥3 loose stools within a 24-hr period)
   2. Vomiting
   3. Stomach cramps
   4. Nausea
   5. Fever
   6. Sore throat
   7. Nasal congestion (e.g., stuffy nose)
   8. Cough with phlegm
   9. Earache or ear infection
   10. Eye infection or eye irritation
   11. Rash or itchy skin
   12. None of the above
7. Do you have any of the following chronic long-term conditions? *(Check all that apply)*
   1. Gastrointestinal problems such as Crohn’s disease or irritable bowel syndrome
   2. Chronic respiratory diseases such as asthma or emphysema
   3. Skin problems such as psoriasis or eczema
   4. Allergies, other than drug allergies
   5. A disease that compromises your immune system, such as cancer, HIV/AIDS, diabetes, or being a transplant recipient
   6. None of the above
8. Did you or anyone in your household at the beach today go swimming or engage in other water activities (e.g., kayaking, windsurfing, paddleboarding) anywhere in the past 14 days?
   1. Yes
   2. No
9. Did you swim or enter the water today?
   1. Yes
   2. No (skip next section)

**Water Activities**

1. Which of the following activities did you or your household members engage in, and who engaged in each activity? *(Check all that apply)*
   1. Swimming
   2. Wading (below your waist)
   3. Surfing
   4. Kitesurfing / kiteboarding
   5. Windsurfing
   6. Wakeboarding
   7. Waterskiing
   8. Paddleboarding
   9. Snorkelling
   10. Underwater diving
   11. Sailing
   12. Boating
   13. Fishing
   14. Canoeing
   15. Kayaking
   16. Rowing / dragon boat racing
   17. Other, specify: ________________
2. When you entered the water today, did you: *(Check all that apply)*
   1. Put your face in the water or submerge your head in the water
   2. Swallow or get any water in your mouth
   3. None of the above

**Beach Activities**

1. While at the beach today, did you: *(Check all that apply)*
   1. Touch algae or seaweed
   2. Apply sunscreen
   3. Apply insect repellent
   4. Eat food at the beach
2. Did you play or dig in the sand today?
3. Yes
4. No (skip next section)

**Sand Activities**

1. Which of the following activities did you engage in today? *(Check all that apply)*
2. Digging in the sand (e.g., building sand castles)
3. Burying yourself in the sand
4. None of the above
5. Did you get sand in your mouth?
6. Yes
7. No

**Other Household Members**

1. Are there any other members of your household with you at the beach today?
2. Yes (generate new instance of individual response part of survey for next household member)
3. No (submit survey)

**Follow-up Questionnaire (7 Days Following Beach Visit)**

**Name**

1. Please enter your first and last name:
2. _______________

We have some questions about each member of your household that attended the beach with you. Please answer the following questions about yourself before answering for other members of your household, answering for each person individually.

**Activities and Symptoms since Beach Visit**

1. After you completed the beach survey with us last week, did you or any other household members participate in any other sand or water activities that day that you didn't already tell us about? (E.g. you told us you didn't go in the water but then decided to swim after completing our survey). If yes, please explain below.
   1. Yes, specify: ________________
   2. No
2. Have you gone swimming or engaged in other water activities (e.g., kayaking, windsurfing, paddleboarding) anywhere since we interviewed you at the beach? E.g., at a beach, pool, splash pad, or wading pool.
3. Yes
4. No
5. Since you were interviewed at the beach, have you had any of the following symptoms? *(Check all that apply)*
   1. Diarrhea (≥3 loose stools within a 24-hr period)
   2. Vomiting
   3. Stomach cramps
   4. Nausea
   5. Fever
   6. Sore throat
   7. Nasal congestion (e.g., stuffy nose)
   8. Cough with phlegm
   9. Earache or ear infection
   10. Eye infection or eye irritation
   11. Rash or itchy skin
   12. None of the above
6. Please select the date when each of your symptoms first started:
   1. Diarrhea (≥3 loose stools within a 24-hr period): _________
   2. Vomiting: _________
   3. Stomach cramps: _________
   4. Nausea: _________
   5. Fever: _________
   6. Sore throat: _________
   7. Nasal congestion (e.g., stuffy nose) : _________
   8. Cough with phlegm: _________
   9. Earache or ear infection: _________
   10. Eye infection or eye irritation: _________
   11. Rash or itchy skin : _________
   12. None of the above

**AGI outcomes**

*(This section will appear only for those who selected AGI-related symptoms)*

1. Did you miss any time from work, school, recreation, or vacation activities because they were sick with diarrhea, vomiting, nausea, and/or stomach cramps, or because you had to care for someone else with these symptoms in your household? If yes, please specify the number of days missed.
   1. Yes, specify number of days missed: _____
2. No
3. Did you take any of the following medications because of your illness with diarrhea, vomiting, nausea, and/or stomach cramps? *(Check all that apply)*
4. Antibiotics or other drugs prescribed by a physician
5. Over-the-counter medications
6. None of the above
7. Did you consult a healthcare provider (e.g., over the phone, in person) about your illness diarrhea, vomiting, nausea, and/or stomach cramps?
   1. Yes
8. No
9. Did you visit an emergency room because of your diarrhea, vomiting, nausea, and/or stomach cramps?
   1. Yes
10. No (skip next question)
11. When you visited the emergency room, were you admitted to a hospital?
12. Yes
13. No

**Other Household Members**

1. Are there any other members of your household who came to the beach with you and completed our beach survey?
2. Yes (generate new instance of survey questions for next household member)
3. No (submit survey)

**Closing Message:**

Thank you for taking the time to participate in this research study! We will notify if you are selected to win one of the e-gift card prizes.

Please follow the link below for more information about beach water quality and how to prevent recreational water illness in Toronto: <http://app.toronto.ca/tpha/beaches.html>
