# Supplementary material for: Recreational water exposures and illness outcomes at a freshwater beach in Toronto, Canada: A prospective cohort pilot study
Source: PLoS One. 2023 Jun 2;18(6):e0286584. doi: 10.1371/journal.pone.0286584 (PMC10237503; doi:10.1371/journal.pone.0286584)
Supplement: S1 Table — (DOCX) [file pone.0286584.s003.docx]

**S1 Table: Comparison of participants who completed the follow-up survey vs. those who did not complete the follow-up survey, Woodbine Beach, Toronto, 2022**

| **Characteristic** | **Yes (N = 287)** | **No (N = 544)** |
| --- | --- | --- |
| Age group (years): |  |  |
| 0-14 | 22 (7.8%) | 81 (15.0%) |
| 15-19 | 50 (17.7%) | 88 (16.3%) |
| 20-39 | 157 (55.5%) | 309 (57.3%) |
| 40+ | 54 (19.1%) | 61 (11.3%) |
| Missing | 4 | 5 |
| Gender identity: |  |  |
| Woman/girl | 195 (69.1%) | 338 (63.1%) |
| Man/boy | 81 (28.7%) | 189 (35.3%) |
| Gender fluid, non-binary, or transgender | 6 (2.1%) | 9 (1.7%) |
| Missing | 5 | 8 |
| Household income (CAD$): |  |  |
| <40,000 | 14 (12.6%) | 48 (21.8%) |
| 40,000-79,999 | 25 (22.5%) | 57 (25.9%) |
| 80,000-149,999 | 40 (36.0%) | 76 (34.5%) |
| 150,000+ | 32 (28.8%) | 39 (17.7%) |
| Missing | 176 | 324 |
| Highest education completed in household: |  |  |
| High school or less | 58 (27.2%) | 128 (34.1%) |
| College, trades, or apprenticeship | 46 (21.6%) | 73 (19.5%) |
| Bachelor’s degree | 65 (30.5%) | 88 (23.5%) |
| Post-graduate degree | 44 (20.7%) | 86 (22.9%) |
| Missing | 74 | 169 |
| Location of residence: |  |  |
| Ontario | 265 (92.3%) | 508 (93.4%) |
| U.S. | 15 (5.2%) | 18 (3.3%) |
| Quebec | 4 (1.4%) | 12 (2.2%) |
| Other provinces | 3 (0.9%) | 6 (1.1%) |
| Ethno-racial identity: |  |  |
| White | 146 (59.3%) | 265 (56.4%) |
| South Asian | 25 (10.2%) | 36 (7.7%) |
| Southeast Asian | 14 (5.7%) | 34 (7.2%) |
| Arab or Middle Eastern | 13 (5.3%) | 34 (7.2%) |
| East Asian | 12 (4.9%) | 29 (6.2%) |
| Latin | 9 (3.7%) | 25 (5.3%) |
| Black | 10 (4.1%) | 23 (4.9%) |
| Multiple ethnicities | 15 (6.1%) | 16 (3.4%) |
| Indigenous | 2 (0.8%) | 8 (1.7%) |
| Missing | 41 | 74 |
| Baseline illness status: |  |  |
| Respiratory illness | 13 (4.5%) | 3 (0.6%) |
| AGI | 3 (1.0%) | 4 (0.7%) |
| Skin infection | 3 (1.0%) | 3 (0.6%) |
| Ear infection | 0 (0.0%) | 1 (0.2%) |
| Eye infection | 0 (0.0%) | 1 (0.2%) |
| Baseline health conditions: |  |  |
| Allergies | 29 (10.1%) | 42 (7.7%) |
| Chronic respiratory condition | 15 (5.2%) | 12 (2.2%) |
| Chronic GI condition | 6 (2.1%) | 9 (1.7%) |
| Immune-compromised | 5 (1.7%) | 5 (0.9%) |
| Engaged in other recreational water activities within the past 2 weeks | 134 (46.7%) | 214 (39.3%) |
| Any water contact at the beach | 180 (62.7%) | 287 (52.8%) |
| Swimming | 141 (49.1%) | 226 (41.5%) |
| Wading (below one’s waist) | 83 (28.9%) | 137 (25.2%) |
| Face contact with water | 66 (23.0%) | 120 (22.1%) |
| Swallowing water | 35 (12.2%) | 59 (10.8%) |
| Any sand contact at the beach | 155 (54.0%) | 311 (57.2%) |
| Digging in the sand | 135 (47.0%) | 271 (49.8%) |
| Burying oneself in the sand | 31 (10.8%) | 104 (19.1%) |
| Sand in mouth | 23 (14.1%) | 84 (26.2%) |
| Contact with algae at the beach | 104 (36.2%) | 208 (38.2%) |
| Applied sunscreen at the beach | 221 (77.0%) | 392 (72.1%) |
| Consumed food at the beach | 202 (70.4%) | 375 (68.9%) |
