# Supplementary material for: Recreational water exposures and illness outcomes at a freshwater beach in Toronto, Canada: A prospective cohort pilot study
Source: PLoS One. 2023 Jun 2;18(6):e0286584. doi: 10.1371/journal.pone.0286584 (PMC10237503; doi:10.1371/journal.pone.0286584)
Supplement: Attachment — Submitted filename: Comments to authors.docx [file pone.0286584.s004.docx]

**Title: Recreational water exposures and illness outcomes at a freshwater beach in Toronto, Canada: a prospective pilot cohort study**

**Manuscript Number: PONE-D-22-34376**

I appreciate the authors for their outstanding team work on such an interesting topic. Their work is novel. I have some suggestion for the authors as mentioned below.

Title: authors would entitle as “Incidence of recreational water activities and illness outcomes at a freshwater beach in Toronto, Canada: a prospective pilot cohort study”

Abstract: The authors would not describe the sentence “Water contact activities were lowest in June (39.1%), increasing in prevalence in July (57.4%) and August (70.8%).” in the abstract section because these findings were not part of their main study objectives. The abstract should include only pertinent findings.

Introduction:

Authors would detail the recreational water activities. What are those activities; how many types; and what are the commonest?

Line- 58: better to say “illnesses”

Authors confined the evidence searches only to Canada and U.S. What is the magnitude and impact of the phenomenon in the other continents?

“The study objectives were to determine characteristics of beachgoers, common water and sand exposures, the incidence of RWI, and the feasibility and recommendations for a larger, national cohort study” It is not clear why the authors included “…recommendations for a larger, national cohort study” to their objective. Recommendation must follow existing result.

Methods and materials

How did the authors identified base line health status was identified? How did authors ruled out previous exposure to bacterial or viral agents? Did authors use a lab-based investigation or used only participants’ verbal report?

What if the participant exposed to viral or bacterial sources (other than RW activities) amid the follow-up period? How did you controlled?

Is your tool validated?

Line-181: “low prevalence”. Is it incidence or prevalence in the eye of cohort study?

In the data analysis section, authors conducted cross-tabulations to compare water and sand exposures by age group, gender identity, highest education level in the household, and ethno-racial identity. However, they didn’t mention any statistical methods, such as chi-square tests or t-est to show differences in each category they used to compare.

Results:

Significant number of participants’ age of was below 16; however, authors mentioned as “To be eligible for participation, at least one household member aged 16 years or older needed to be present to conduct the survey in English” Can you explain this issue, please?

Discussion:

Authors reported that “parents and caregivers were often reluctant to take time to participate and complete surveys on the beach given their childcare duties, contributing to the responses of ‘not wanting to be bothered’ and ‘not having time” but they didn’t mentioned the average time each interview took.

Authors would limit their discussion to their research objectives. Most of the discussion, particularly the first and the second paragraphs, are not based on the objectives.
