# Supplementary material for: Recreational water exposures and illness outcomes at a freshwater beach in Toronto, Canada: A prospective cohort pilot study
Source: PLoS One. 2023 Jun 2;18(6):e0286584. doi: 10.1371/journal.pone.0286584 (PMC10237503; doi:10.1371/journal.pone.0286584)
Supplement: Attachment — Submitted filename: Response to reviewers.docx [file pone.0286584.s006.docx]

**PONE-D-22-34376R1: Response to Reviewers**

**********************************************

**Editor Comments:**

1) The authors conclude that a larger national cohort study is needed, but they do not address one finding of the pilot study- namely at the site they studied, the water was likely to be much too clean to see observable health relationships. Any larger study should encompass a wider range of water quality, including sites that do not have excellent water quality and sites that are impacted by sources of human sewage which are the riskiest for beachgoers. Also note that this study may be considerably expensive so the benefits should outweigh the costs

*We agree with these points – we had a brief statement about this at the end of the limitations section, but have moved this earlier in the discussion where we discuss the water quality results, and have expanded on it accordingly.*

2) This study only considered limited measures of water quality, there are many advancement in water quality testing including human markers, rapid methods, and viral indicators. Any large national study should consider these as well

*This is also a good point and we have added this to our suggestions for follow-up research in the discussion.*

3) The conclusion that routine monitoring of sand quality should be considered based on this study seems premature- simply because more people are in contact with sand does not necessarily mean it should be monitored which could be considerably expensive, and may come at the trade off of other types of water quality testing. I do not know anywhere where sand is currently regularly monitored, although WHO provided a provisional value, this has not really been tested or validated broadly. Please reconsider this recommendation, at least until results of a larger study provide more conclusive evidence

*We have revised this recommendation as suggested, to instead suggest that further research is needed on possible sand contamination and its role in causing RWI. See the modified paragraph in the discussion and revised statement in the conclusion.*

*Note that the reference list has been slightly altered to accommodate a few additional articles cited as part of this revised discussion, and to update bibliographic details of a couple of the other references.*
